# Absence of gut microbiota reduces neonatal survival and exacerbates liver disease in *Cyp2c70*-deficient mice with a human-like bile acid composition

**DOI:** 10.1042/CS20230413

**Published:** 2023-07-14

**Authors:** Wilhelm Sjöland, Annika Wahlström, Kassem Makki, Marc Schöler, Antonio Molinaro, Lisa Olsson, Thomas Uwe Greiner, Robert Caesar, Jan Freark de Boer, Folkert Kuipers, Fredrik Bäckhed, Hanns-Ulrich Marschall

**Affiliations:** 1 Wallenberg Laboratory and Department of Molecular and Clinical Medicine, Institute of Medicine, Sahlgrenska Academy, University of Gothenburg, S-413 45 Gothenburg, Sweden; 2 Region Västra Götaland, Sahlgrenska University Hospital, Department of Medicine, Gothenburg, Sweden; 3 Department of Pediatrics, University of Groningen, University Medical Center Groningen, The Netherlands; 4 Department of Laboratory Medicine, University of Groningen, University Medical Center Groningen, Groningen, the Netherlands; 5 European Research Institute for the Biology of Ageing (ERIBA), University of Groningen, University Medical Center Groningen, Groningen, the Netherlands; 6 Region Västra Götaland, Sahlgrenska University Hospital, Department of Clinical Physiology, Gothenburg, Sweden; 7 Novo Nordisk Foundation Center for Basic Metabolic Research, Faculty of Health Sciences, University of Copenhagen, Copenhagen, DK-2200, Denmark

**Keywords:** bile acids, cyp2c70, faecal microbiota transplantation, gut microbiota, hepatobiliary disease, ursodeoxycholic acid

## Abstract

Mice with deletion of *Cyp2c70* have a human-like bile acid composition, display age- and sex-dependent signs of hepatobiliary disease and can be used as a model to study interactions between bile acids and the gut microbiota in cholestatic liver disease. In the present study, we rederived *Cyp2c70*^−/−^ mice as germ-free (GF) and colonized them with a human or a mouse microbiota to investigate whether the presence of a microbiota can be protective in cholangiopathic liver disease associated with *Cyp2c70-*deficiency. GF *Cyp2c70*^−/−^ mice showed reduced neonatal survival, liver fibrosis, and distinct cholangiocyte proliferation. Colonization of germ-free breeding pairs with a human or a mouse microbiota normalized neonatal survival of the offspring, and particularly colonization with mouse microbiota from a conventionally raised mouse improved the liver phenotype at 6–10 weeks of age. The improved liver phenotype in conventionalized (CD) *Cyp2c70*^−/−^ mice was associated with increased levels of tauro-ursodeoxycholic acid (TUDCA) and UDCA, resulting in a more hydrophilic bile acid profile compared with GF and humanized *Cyp2c70*^−/−^ mice. The hydrophobicity index of biliary bile acids of CD *Cyp2c70*^−/−^ mice was associated with changes in gut microbiota, liver weight, liver transaminases, and liver fibrosis. Hence, our results indicate that neonatal survival of *Cyp2c70*^−/−^ mice seems to depend on the establishment of a gut microbiota at birth, and the improved liver phenotype in CD *Cyp2c70*^−/−^ mice may be mediated by a larger proportion of TUDCA/UDCA in the circulating bile acid pool and/or by the presence of specific bacteria.

## Introduction

Interactions between bile acids and the gut microbiota affect host physiology and metabolism and may influence the development and progression of liver disease, but underlying mechanisms are still unknown. The use of mouse models to study the cross-talk between bile acids and the gut microbiota is limited by differences in bile acid profile between mice and humans. In addition to the human primary bile acids cholic acid (CA) and chenodeoxycholic (CDCA), mice also synthesize very hydrophilic 6β-hydroxylated primary bile acids such as α-muricholic acid (αMCA) and β-muricholic acid (βMCA) [1,2]. CYP2C70 is the enzyme responsible for the formation of 6β-hydroxylated murine bile acids [3] and mice with deletion of the *Cyp2c70* gene have a human-like bile acid pool and may be a more relevant model to study bile acid metabolism [4,5]. *Cyp2c70* knockout mice display increased hepatic inflammation, fibrosis and transient neonatal cholestasis [6] and could therefore constitute a model for cholestatic liver disease such as primary sclerosing cholangitis (PSC), in which interactions between gut microbiota and bile acids have been implicated [7,8]. Similarly, studies with multidrug resistance gene 2 (*Mdr2* or *Abcb4*)-deficient mice, another mouse model of genetically induced cholestatic liver disease, have shown that the absence of a gut microbiota worsens cholestasis and liver phenotype [9] and leads to increased lethality [10]. Thus, we have previously suggested that the gut microbiota might have a protective role in cholestatic liver diseases [11]. Therefore, rederiving *Cyp2c70*-deficient mice as germ-free (GF) and colonizing them with a human microbiota may constitute a more physiological model to investigate how the gut microbiota interacts with bile acids to modulate liver disease.

## Methods

### Animal experiments

#### Generation of GF Cyp2c70^−/−^ mice and colonization experiment

Conventionally raised wild-type C57/Bl6 females were crossed with conventionally raised *Cyp2c70*^−/−^ (C57Bl/6J-Cyp2c70^em3Umcg^) males provided by Folkert Kuipers’ lab in Groningen, The Netherlands (www.labpediatricsrug.nl). *Cyp2c70*^+/−^ mice were rederived as GF by caesarian-section. The uterine packages from pregnant donor mice were dissected and transferred into a sterile isolator via a transfer chamber filled with chlorine bleach. The pups were removed from the uterine package and placed in a cage with a GF Swiss Webster dam. The GF *Cyp2c70^+/−^* mice were then housed in sterile isolators and crossed to generate GF *Cyp2c70*^−/−^, *Cyp2c70*^+/−^, and *Cyp2c70*^+/+^ mice. For humanization, GF *Cyp2c70*^+/−^ breeding pairs were transferred to a new isolator and colonized with fresh human faeces from a 47-year-old healthy (no medications or nutritional supplements) male donor. For conventionalization, GF *Cyp2c70*^+/−^ breeding pairs were transferred to our open facility and colonized with caecal content from a conventionally raised wild-type C57/Bl6 mouse. Colonization was performed by diluting the human faecal or mouse caecal samples (approximately 0.5 g) in 5 ml reduced PBS containing 0.2 g/l Na_2_S and 0.5 g/l cysteine as reducing agents in Hungate tubes. About 0.2 ml of this suspension was introduced by gavage into each GF mouse. The samples were obtained shortly before colonization and immediately (within 5 min) diluted into the PBS and introduced into the GF mice within 2 h after dilution. The humanized (HUM) mice were gavaged a second time after two days with inoculum from a new fresh faecal sample from the same human donor.

The GF and the HUM mice were maintained in flexible plastic gnotobiotic isolators under a strict 12 h light cycle and fed an autoclaved chow diet (LabDiet 5021, St Louis, MO) *ad libitum*. GF isolators were routinely tested for sterility by culturing and PCR analysis of faeces amplifying the 16S rRNA gene. The conventionalized (CD) mice were maintained in conventional cages under a strict 12 h light cycle and fed the same autoclaved chow diet as the GF and HUM mice *ad libitum*.

All animal experiments were performed at Experimental Biomedicine, University of Gothenburg, using protocols approved by the Gothenburg Animal Research Ethics Committee (Ethical number 4805/23).

#### Tissue collection

Blood was collected from the inferior vena cava under deep isoflurane-induced anesthesia following 4 h fasting. The mice were euthanized by cervical dislocation and tissues (liver, gallbladder, and caecum) were collected and immediately frozen in liquid nitrogen and stored at −80°C until further processing. In addition, liver biopsies were fixed in 4% paraformaldehyde at 4°C for at least 48 h and then washed with PBS and stored in 70% ethanol at 4°C until paraffine-embedding.

### Histology

Liver sections (5 µm) were processed as previously described [12]. Sections were stained for collagen using Sirius Red (Sigma-Aldrich, 366548) and analyzed with Python (3.9.15) using Napari (0.4.17), pyclesperanto (0.22.0), SimpleITK (2.2.1) [13], and APOC (0.12.0) using the devbio-napari (0.8.1) package. Images were acquired with a 10× objective on a Zeiss Apotome Axioplan 2. For Sirius Red quantification, random forest decision trees were trained on an image and applied to all images. Labeled pixels were quantified and the total pixel count was normalized to the image resolution. Labeled pixels were mapped to an image for manual control of segmentation.

### Immunohistochemistry

Liver sections were stained for cytokeratin 19 (CK19) using a rabbit antibody (Abcam, ab-52625, 1:300). The sections were incubated overnight at 4°C followed by staining using a secondary Goat Anti-Rabbit Alexa 594 antibody (Thermo Fischer Scientific, A-11072, 1:300). The sections were stained for nuclei using Hoechst solution (1:5000). Images were acquired with a 10× objective using a Nikon Eclipse Ni-E.

Images of sections stained with CK19 antibody were analyzed with Python (3.9.15) using Napari (0.4.17), pyclesperanto (0.22.0) and SimpleITK (2.2.1) using the devbio-napari (0.8.1) package. More specifically, a Top-hat filter was applied to remove background using default settings, and a median filter was applied for noise reduction using default settings. Stained areas were labeled using the Renyi entropy threshold. The labeled pixels were quantified, and the total pixel count was normalized to the image resolution. Labeled pixels were mapped to an image for manual control of labeling.

### Liver function tests

Liver transaminases, aspartate transaminase (AST), and alanine transaminase (ALT) were measured in 100 µl of serum from the inferior caval vein. Analyses were performed at Clinical Chemistry Department, Sahlgrenska University Hospital, using the system Alinity ci 1303, software version 3.4.0 (Abbott laboratories).

### Quantitative real-time PCR

Approximately 30 mg of liver was homogenized using Tissuelyser II (Qiagen) and total RNA was isolated using RNeasy mini kit (Qiagen, 74106). High-Capacity cDNA Reverse Transcription Kit (Applied Biosystems, 4368813) was used to synthesize 20 µl cDNA templates from 500 ng purified RNA using random hexamer primers, and the products were diluted 7x before use in subsequent reactions. 1× iQ™ SYBR® Green Supermix (Bio-Rad, 1708886) was used for qRT-PCR at final reaction volumes of 10 µl. 900 nM gene-specific primers were used in each reaction, and all gene expression data were normalized to TATA-binding protein (*Tbp*) gene expression (forward: 5′-CGA ACA GAG TTT GAC AGA GA-3′; reverse: 5′-CAT CTT CAC CAG GAT CAG C-3′). Analyzed genes and primers were chemokine ligand 2 (*Ccl2*) (forward: 5′-CCC CAA GAA GGA ATG GGT CC-3′; reverse: 5′-GGT TGT GGA AAA GGT AGT GG-3′), neutrophil membrane glycoprotein NB1 (*Cd177*) (forward: 5′-CCG GAC AGG GAC TTC TGT AA-3′; reverse: 5′-ACA CAG CTG CCG GTT GAA-3′), adhesion G protein-coupled receptor E1 (*Adgre1* or *F4/80*) (forward: 5′-CTT TGG CTA TGG GCT TCC AGT C-3′; reverse: 5′-GCA AGG AGG ACA GAG TTT ATC GTG-3′), and tumor necrosis factor *α* (*Tnfα*) (forward: 5′-CCC CAA AGG GAT GAG AAG TT-3′; reverse: 5′-CTC CTC CAC TTG GTG GTT TG-3′). Assays were performed in a CFX-Connect Real-Time System (Bio-Rad), and data were analyzed using the ΔΔCT analysis method.

### Bile acid analysis

Gallbladder, liver, caecum, and serum from the inferior caval vein were collected for bile acid analysis. Tissue samples (∼50–100 mg) and the whole gallbladder were extracted with methanol, containing d4-TCA, d4-CA, d4-CDCA, and d4-LCA (2.5 µM of each) as internal standards. The samples were homogenized with ceramic beads for 10 min using the Tissuelyser II (Qiagen). After 10 min of centrifugation at 20,000 *** g***, 20 μl of supernatant was diluted with 980 μl of methanol:water 1:1. Serum samples (25 µl) were extracted with 10 volumes of methanol containing 50 nM of respective deuterated internal standards. After 5 min of vortex and 10 min of centrifugation at 20 000 ***g***, supernatants were evaporated using a stream of nitrogen and reconstituted in 100 µl of methanol: water [1:1]. Approximately 5 µl of the samples were used for the bile acid analysis.

Bile acids were analyzed using ultra-performance liquid chromatography-tandem mass spectrometry (UPLC-MS/MS). After injection the bile acids were separated on a C18 column (1.7 µm, 2.1 × 100 mm; Kinetex, Phenomenex, U.S.A.) using water with 7.5 mM ammonium acetate and 0.019% formic acid (mobile phase A) and acetonitrile with 0.1% formic acid (mobile phase B). The chromatographic separation started with 1-min isocratic separation at 20% B. The B-phase was then increased to 35% for 4 min. During the next 10 min, the B-phase was increased to 100%, and it was held at 100% for 3.5 min before returning to 20%. The total runtime was 20 min. Bile acids were detected using multiple reaction monitoring (MRM) in negative mode on a QTRAP 5500 mass spectrometer (Sciex, Concord, Canada), and quantification was made against appropriate deuterated internal standards with adjustments by using external individual standard curves. Heuman’s method was used to determine hydrophobicity indices of biliary bile acids [14].

### 16S rRNA microbiota analysis

Genomic DNA was extracted using the Nucleospin Soil Kit (Macherey-Nagel, 740780.50). Mouse caecum (∼50–100 mg) was aliquoted and extracted using two rounds of chemical lysis and bead-beating. For each round, samples were put into SL2 buffer with SX enhancer and subsequently incubated at 90°C for 10 min. The samples were then sheared for three runs of bead beating at 5.5 m/s for 60 s in a FastPrep-24 Instrument (MP Biomedicals) with 5 min on ice in between. The V4 region of the 16S rRNA gene was amplified with a PCR program of denaturation for 3 min at 94°C, with 25 cycles of denaturation for 45 s at 94°C, annealing for 60 s at 52°C, and elongation for 90 s at 72°C, concluding with elongation for 10 min at 72°C. Reaction volumes were 25 μl and contained 200 nM of 515F and 806R primers [15] with added 1× 5PRIME HotMasterMix (5PRIME), 0.4 mg/ml BSA, and 5% dimethylsulfoxide, and duplicate reactions were run. Duplicate reactions were combined and Quant-iT PicoGreen dsDNA kit (Invitrogen, P11496) was used to determine concentration of DNA. The NucleoSpin Gel and PCR Clean-Up kit (Macherey-Nagel, 740609.250) was used for purification of the combined reactions and samples were subsequently diluted to 10 ng/µl and pooled in equal amounts. The pools were repurified with AMPure XP Kit (Agencourt, A63880) for removal of primer-dimers and short products. The repurified pools were run on a 2100 Bioanalyzer (Agilent) to certify that short amplification product was not present. The pooled amplicons were sequenced on an in-house MiSeq instrument (RTA (v.1.18.54.0) with MCS (v.2.6.2.1; Illumina) with the V2 kit (2 × 250 bp paired-ended reads; Illumina).

Reads were merged, filtered, de-duplicated, de-noised and aligned to Zero-radius operational taxonomic units (zOTUs) using Usearch 11.0.067 [16]. During merging a maximum of five mismatches were allowed in the alignment with a merge length between 251 and 257. Merged reads were filtered and reads with > 1 expected error were excluded, and remaining reads were subsequently de-duplicated. The filtered reads were de-noised using the UNOISE3 algorithm [17], and reads were aligned to each zOTUs’ sequence concluding pre-processing with Usearch. Each zOTUs’ sequence was then taxonomically assigned using DADA2's assignTaxonomy(minBoot = 50) [18,19] with a trained SILVA database v. 138 [20,21]. Prior to analysis, zOTUs that contributed less than 0.002% of the total amount of reads were filtered out and remaining zOTUs were normalized by resampling with replacement so that all samples had the same library size.

Microbiota analysis was performed using the software R (4.2.2). The phyloseq package (1.42.0) was used for handling zOTU data [22]. Correlation analysis was performed on zOTUs aggregated at the lowest taxonomic rank they were annotated at using Spearman’s correlation and visualized with either ggplot (3.4.0) or the ComplexHeatmap package (2.14.0). Prior to correlation analysis the zOTU counts were first centered log-ratio transformed filling zeroes with half of the minimum value. Principal Coordinate Analysis (PCoA) was performed using weighted UniFrac distance matrix and was also used for all other distance-based methods. Microbiota composition dissimilarity was tested with 9999 permutations with the adonis2 function from the vegan package (2.6.4) [23].

### Statistical analyses

Mouse physiology data is presented with bar plots ± SEM and tested with unpaired two-tailed Student’s t-test. Genotype ratios were tested against a Mendelian ratio (1:2:1) with a Chi-square goodness of fit test. Comparisons of survival curves were made using Log-rank (Mantel-Cox) tests. Comparisons of the degree of Sirius Red and CK19 staining were made between *Cyp2c70*^+/+^ and *Cyp2c70*^−/−^ females and males separately and only within the same group (GF, HUM and CD) and tested with Wilcoxon rank sum tests. Bile acid data are presented with pie-charts with bile acids contributing to ≥3% of the mean of the sum of bile acids. Comparisons of bile acid concentrations were made between *Cyp2c70*^+/+^ and *Cyp2c70*^−/−^ females and males separately and only within the same group (GF, HUM and CD) and tested with Wilcoxon rank sum tests using Benjamini and Hochberg correction for multiple testing adjustment of individual bile acids. Differences in biliary hydrophobicity indices were tested using Kruskal–Wallis H tests. Following rejection of the null hypothesis, the Conover–Iman test was used for multiple comparisons using Benjamini and Hochberg correction for multiple testing adjustment. Differences in gene expression and liver transaminases were tested with Wilcoxon rank sum tests, and comparisons were made between *Cyp2c70*^+/+^ and *Cyp2c70*^−/−^ females and males separately and only within the same group (GF, HUM and CD). Correlations between *Cd177*, *F4/80, Tnfα*, *Ccl2*, AST, or ALT and biliary hydrophobicity indices of bile acids were performed using Spearman’s correlation. Differences in α-diversity were tested with Wilcoxon rank sum tests between *Cyp2c70*^+/+^ and *Cyp2c70*^−/−^ females and males separately and only within the same group (HUM and CD). Differences in abundance of *Desulfovibrio* and *P. excrementihominis* were tested with Wilcoxon rank sum tests and comparisons were made between early and late timepoints in CD *Cyp2c70*^−/−^ mice. Correlations between bacterial taxa and biliary hydrophobicity indices of bile acids were tested using Spearman’s correlation and adjusted for multiple testing using Benjamini and Hochberg correction.

## Results

### Deletion of *Cyp2c70* is partially lethal in the absence of a gut microbiota

**Figure 1:**
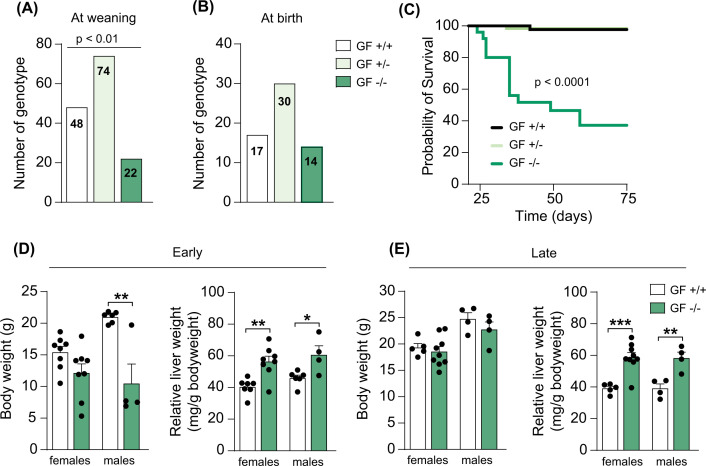
Reduced survival and increased relative liver weight in GF *Cyp2c70*^−/−^ mice. (**A,B**) Number of GF mice genotyped at weaning (three weeks of age) (**A**) and immediately after birth (**B**). (**C**) Kaplan–Meier estimator of survival over time in GF *Cyp2c70* mice (*Cyp2c70*^+/+^, *n*=46, *Cyp2c70*^+/−^, *n*=61, *Cyp2c70*^−/−^, *n*=25) analyzed with Log-rank (Mantel-Cox) test. (**D,E**) Body weight and relative liver weight in GF *Cyp2c70*^+/+^ and *Cyp2c70*^−/−^ mice at the early timepoint (**D**) (*n*=4–8 mice per group) and the late timepoint (**E**) (*n*=4–9 mice per group). Data are presented as mean ± SEM, *P<0.05, **P<0.01, ***P<0.001 indicate differences between female or male GF *Cyp2c70*^+/+^ and GF *Cyp2c70*^−/−^ mice analyzed with two-tailed unpaired *t*-tests.

We rederived *Cyp2c70*^+/−^ mice as GF by caesarian-section and bred GF *Cyp2c70*^+/−^ females with *Cyp2c70*^+/−^ males to obtain GF *Cyp2c70*^−/−^ mice and observed that the offspring did not display the expected Mendelian ratio (1:2:1) at three weeks of age. From 144 pups, we obtained 48 *Cyp2c70*^+/+^ mice, 74 *Cyp2c70*^+/−^ mice and 22 *Cyp2c70*^−/−^ mice, indicating that complete deletion of the *Cyp2c70* gene affects survival in a GF setting (Figure 1A). No differences were found between females and males. To investigate if the detrimental effects of *Cyp2c70* deletion occur *in utero* or if the mice are actually born at the expected Mendelian ratio, we set up new breeding pairs and genotyped 9 litters at birth. From 61 pups, we obtained 17 *Cyp2c70*^+/+^ mice, 30 *Cyp2c70*^+/−^ mice, and 14 *Cyp2c70*^−/−^ mice, which corresponded to a Mendelian ratio (Figure 1B). This indicated that the reduced number of *Cyp2c70*^−/−^ mice at 3 weeks was due to mortality during the neonatal period and not *in utero*. We then assessed overall survival in a subset of mice, from day 21 until day 75, and found that the median survival of the GF *Cyp2c70*^−/−^ mice was 49 days (Figure 1C). Importantly, mice displaying severe worsening of the general condition such as weakness, rugged fur, and passivity were euthanized and considered not viable. Of note, the median survival for GF *Cyp2c70*^+/+^ or *Cyp2c70*^+/−^ littermate controls could not be determined since only one mouse in each group had died during this period.

Next, to further investigate the impact of *Cyp2c70* deletion under GF conditions we euthanized *Cyp2c70*^−/−^ mice and their *Cyp2c70*^+/+^ littermate controls to evaluate phenotypical parameters. We euthanized mice at an early timepoint, i.e., 4–5 weeks of age, and at a later timepoint, i.e., at 6–10 weeks of age. At the early timepoint, most of the *Cyp2c70*^−/−^ mice were small and hunchbacked. Furthermore, male *Cyp2c70*^−/−^ mice had a lower body weight and both female and male *Cyp2c70*^−/−^ mice showed an increased relative liver weight compared with their *Cyp2c70*^+/+^ littermate controls (Figure 1D). At the later timepoint, there were no differences in body weight anymore, but the relative liver weights were still significantly higher in both female and male *Cyp2c70*^−/−^ mice compared with their *Cyp2c70*^+/+^ littermate controls (Figure 1E).

### Colonization with a human or a mouse microbiota improves neonatal survival

**Figure 2:**
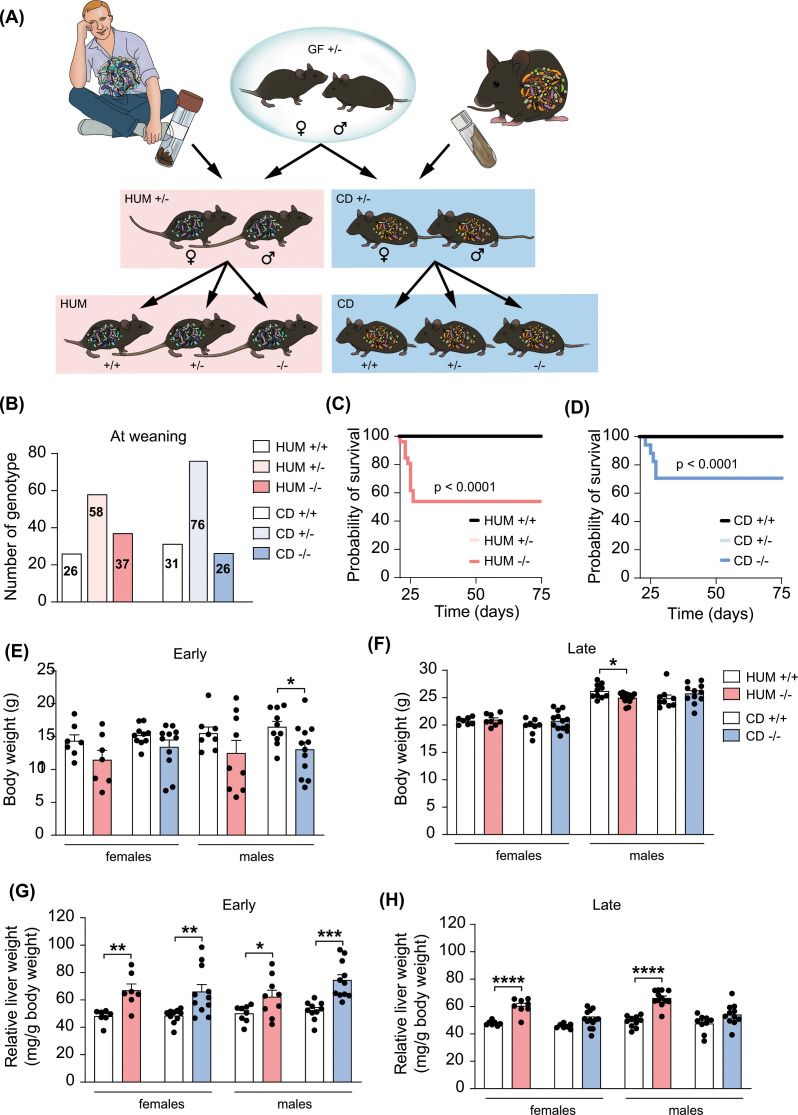
Colonization of *Cyp2c70^+/−^* mice with human or mouse microbiota (**A**) Graphical illustration of the experimental design for the colonization experiments. (**B**) Number of HUM and CD mice genotyped at weaning (3 weeks of age). (**C,D**) Kaplan–Meier estimator of survival over time in HUM mice (**C**) (*Cyp2c70*^+/+^, *n*=21; *Cyp2c70*^+/−^, *n*=45; *Cyp2c70*^−/−^, *n*= 26) and CD *Cyp2c70* mice (**D**) (*Cyp2c70*^+/+^, *n*=25, *Cyp2c70*^+/−^, *n*=37, *Cyp2c70*^−/−^, *n*=17) analyzed with Log-rank (Mantel-Cox) tests. (**E,F**) Body weight in HUM and CD *Cyp2c70*^+/+^ and *Cyp2c70*^−/−^ mice at the early timepoint (**E**) (HUM *n*=7–9 mice per group, CD *n*=8–11 mice per group) and the late timepoint (**F**) (HUM *n*=7–9 mice per group, CD *n*=8–13 mice per group). (**G,H**) Relative liver weight of HUM and CD *Cyp2c70*^+/+^ and *Cyp2c70*^−/−^ mice at the early timepoint (**G**) (same mice as in panel E) and the late timepoint (**H**) (same mice as in panel F). Data are presented as mean ± SEM; *P<0.05, **P<0.01, ***P<0.001, ****P<0.0001 indicate differences between female or male *Cyp2c70*^+/+^ and *Cyp2c70*^−/−^ mice within each group (HUM or CD) analyzed with two-tailed unpaired t-tests.

To test our hypothesis that a gut microbiota can improve survival and the hepatobiliary phenotype in *Cyp2c70*^−/−^ mice, we colonized mice with human or mouse microbiota. First, *Cyp2c70*^+/−^ mice were transferred into a new isolator and colonized with human microbiota from a healthy donor and 5 breeding pairs were set up to generate humanized (HUM) *Cyp2c70*^−/−^ mice and HUM *Cyp2c70*^+/+^ littermate controls (Figure 2A). We obtained four to five litters from each breeding pair, generating 121 pups in total. Genotyping at three weeks showed the following distribution: 26 HUM *Cyp2c70*^+/+^, 58 HUM *Cyp2c70*^+/−^ and 37 HUM *Cyp2c70*^−/−^ mice (Figure 2B), which corresponds to a Mendelian ratio, indicating that there was no increased mortality during the first three neonatal weeks. However, a subset of the *Cyp2c70*^−/−^ mice died or needed to be euthanized between day 21 and 26; thus, the mortality was not completely eliminated (Figure 2C).

Next, to generate conventionalized (CD) mice, we transferred GF *Cyp2c70*^+/−^ mice into our barrier facility and colonized them with caecal content from a conventionally raised mouse and set up three breeding pairs to obtain CD *Cyp2c70*^−/−^ mice (Figure 2A). The genotype distribution at 3 weeks was: 31 CD *Cyp2c70*^+/+^, 76 CD *Cyp2c70*^+/−^, and 26 CD *Cyp2c70*^−/−^ mice, which is consistent with the expected Mendelian ratio (Figure 2B). Similar to HUM mice, the overall survival of CD *Cyp2c70*^−/−^ mice was improved (Figure 2D).

### Colonization with a mouse microbiota ameliorates the liver phenotype of *Cyp2c70*^−/−^ mice

HUM and CD mice were euthanized at similar timepoints as the GF mice. At the early timepoint, male CD *Cyp2c70*^−/−^ mice had a decreased body weight compared with their CD *Cyp2c70*^+/+^ littermate controls (Figure 2E) while genotype did not affect body weight in CD females or HUM mice. At the later timepoint, body weight was slightly lower in male HUM *Cyp2c70*^−/−^ mice compared to their HUM *Cyp2c70*^+/+^ littermate controls (Figure 2F) whereas there were no differences in HUM females or CD mice. Relative liver weight was increased in both HUM and CD *Cyp2c70*^−/−^ mice compared with their *Cyp2c70*^+/+^ counterparts at the early timepoint (Figure 2G), while at the later timepoint, relative liver weight was still higher in HUM *Cyp2c70*^−/−^ mice, but not in the CD *Cyp2c70*^−/−^ mice (Figure 2H).

**Figure 3:**
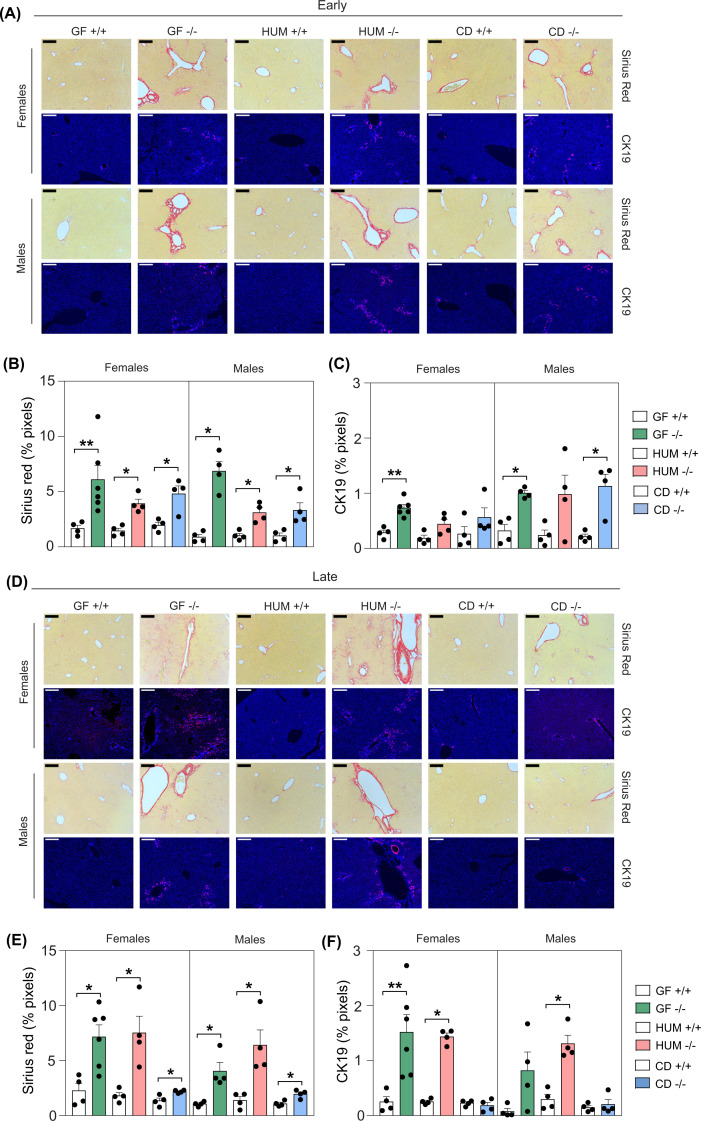
Ameliorated liver phenotype in CD *Cyp2c70^−/−^* mice (**A–F**) Histological and immunological staining and quantification showing Sirius Red and CK19 in livers from female and male GF, HUM and CD *Cyp2c70*^+/+^ and *Cyp2c70*^−/−^ mice at the early timepoint (**A–C**) and at the late timepoint (**D–F**). 200 µm indicators are shown. To increase visibility and brightness for the visual presentation of CK19-stained area the γ was set to 0.8 and gain to 1.7 (Blue: Hoechst, Red: CK19). Data are presented as mean ± SEM; *P<0.05 and **P<0.01 indicate differences between female or male *Cyp2c70*^+/+^ and *Cyp2c70*^−/−^ mice within each group (GF, HUM or CD) analyzed with Wilcoxon rank sum tests.

Sirius Red and CK19 staining showed that GF, HUM and CD *Cyp2c70*^−/−^ mice had increased liver fibrosis and cholangiocyte proliferation at the early timepoint (Figure 3A–C). At the later timepoint, there were still clear features of liver fibrosis and cholangiocyte proliferation in GF and HUM *Cyp2c70*^−/−^ mice, but not in CD *Cyp2c70*^−/−^ mice (Figure 3D–F). In addition, qPCR analyses of liver tissue showed increased expression of immune cell (*Cd177* and *F4/80*) and inflammation (*Tnfα* and *Ccl2*) markers in GF, HUM and CD *Cyp2c70*^−/−^ mice at the early time point (Supplementary Figure S1A). At the late timepoint, there were still increased levels of infiltration and inflammation in the GF and HUM *Cyp2c70*^−/−^ mice, while the levels in the CD *Cyp2c70*^−/−^ mice were similar to CD *Cyp2c70*^+/+^ mice, which is consistent with an improved liver phenotype. Liver function tests in serum showed elevated liver transaminases (AST and ALT) in GF and HUM *Cyp2c70*^−/−^ mice at both timepoints and the CD *Cyp2c70*^−/−^ mice showed increased levels at the early timepoint, but normalized levels at the late timepoint mainly in the male mice (Supplementary Figure S1B).

In summary, colonization with a mouse or human microbiota improved neonatal survival of *Cyp2c70*^−/−^ mice, and the presence of a mouse microbiota also ameliorated the liver phenotype at 6–10 weeks of age.

### Colonization with a mouse microbiota increases production of TUDCA/UDCA

**Figure 4:**
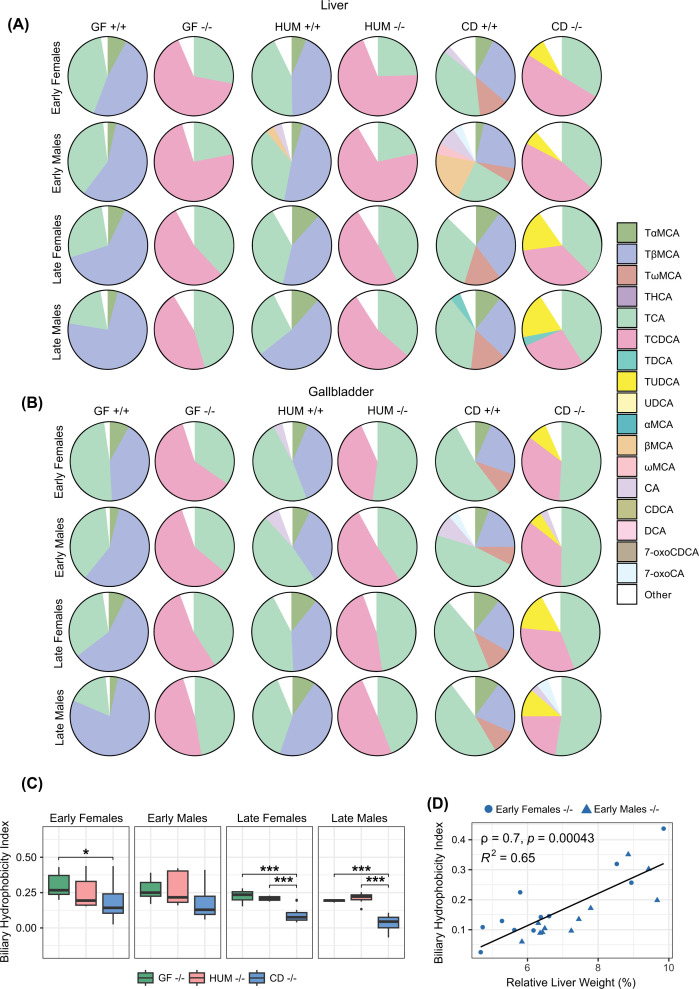
Bile acid profiles in liver and gallbladder from GF, HUM, and CD mice (**A,B**) Bile acid profiles in liver (**A**) and gallbladder (**B**) from female and male GF, HUM, and CD *Cyp2c70*^+/+^ and *Cyp2c70^−^*^/−^ mice at early and late timepoints. (**C**) Biliary hydrophobicity index of female and male GF, HUM, and CD *Cyp2c7*0^−/−^ mice at the early and late timepoints presented with a box plot. The lower (quartile 1) and upper (quartile 3) quartiles form the box with median represented by the central line. Whiskers reach to 1.5× the inner quartile range. Outliers are presented with visible points beyond the whiskers. (**D**) Scatterplot with a linear model fit and Spearman’s correlation analysis of biliary hydrophobicity index, and relative liver weight in CD *Cyp2c70*^−/−^ mice at the early timepoint. *P<0.05, **P<0.01, ***P<0.001 indicate differences between female or male GF, HUM and CD *Cyp2c70*^−/−^ mice analyzed with Kruskal–Wallis *H* tests with the Conover-Iman test as post hoc using Benjamini and Hochberg correction for multiple testing adjustment. Females, *n* = 5–12 mice per group; males, *n* = 4–12 mice per group. αMCA, α-muricholic acid; βMCA, β-muricholic acid; ωMCA, ω-muricholic acid; 7-oxoCDCA, 7-oxochenodeoxycholic acid; 7-oxoCA, 7-oxocholic acid; CA, cholic acid; CDCA, chenodeoxycholic acid; DCA, deoxycholic acid; TαMCA, tauro-α-muricholic acid; TβMCA, tauro-β-muricholic acid; TωMCA, tauro-ω-muricholic acid; TCA, tauro-cholic acid; TCDCA, tauro-chenodeoxycholic acid; TDCA, tauro-deoxycholic acid; THCA, tauro-hyocholic acid; TUDCA, tauro-ursodeoxycholic acid; UDCA, ursodeoxycholic acid.

Bile acid analyses showed that deletion of *Cyp2c70* decreased the levels of primary murine bile acids, TαMCA/αMCA and TβMCA/βMCA, and increased the proportion of TCDCA/CDCA in liver and gallbladder, resulting in a human-like primary bile acid profile (Figure 4A,B and Supplementary Table S1A,B). These observations are in line with previous findings [4–6,24]. We found traces of murine bile acids in some of the *Cyp2c70*^−/−^ mice. This has previously been observed and may be explained by the fact that mice are coprophagous and *Cyp2c70*^−/−^ mice were co-housed together with their *Cyp2c70*^+/+^ littermates [6].

**Figure 5:**
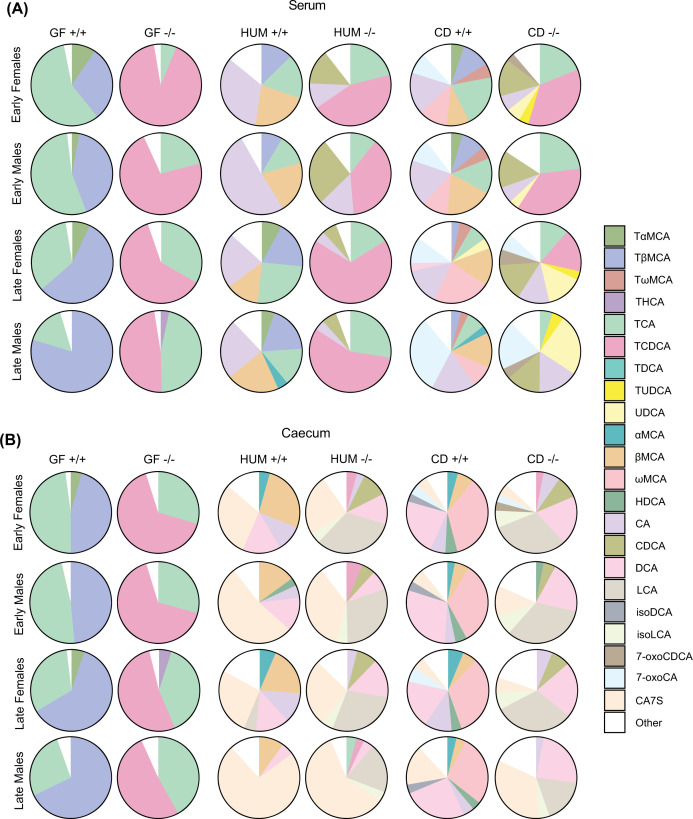
Bile acid profiles in serum and caecum from GF, HUM, and CD mice (**A,B**) Bile acid profiles in serum (**A**) and caecum (**B**) from female and male GF, HUM, and CD *Cyp2c70*^+/+^ and *Cyp2c70^−^*^/−^ mice at the early and late timepoint. Females, *n*=4–13 mice per group; males, *n*=4–13 mice per group. αMCA, α-muricholic acid; βMCA, β-muricholic acid; ωMCA, ω-muricholic acid; 7-oxoCDCA, 7-oxochenodeoxycholic acid; 7-oxoCA, 7-oxocholic acid; CA7S, cholic acid-7-sulfate; CA, cholic acid; CDCA, chenodeoxycholic acid; DCA, deoxycholic acid; HDCA, hyodeoxycholic acid; isoDCA, iso-deoxycholic acid (3β-deoxycholic acid); isoLCA, iso-lithocholic acid (3β-lithocholic acid); LCA, lithocholic acid; TαMCA, tauro-α-muricholic acid; TβMCA, tauro-β-muricholic acid; TωMCA, tauro-ω-muricholic acid; TCA, tauro-cholic acid; TCDCA, tauro-chenodeoxycholic acid; TDCA, tauro-deoxycholic acid; THCA, tauro-hyocholic acid; TUDCA, tauro-ursodeoxycholic acid; UDCA, ursodeoxycholic acid.

In GF *Cyp2c70*^−/−^ mice, bile acid profiles in liver, gallbladder, serum and caecum contained almost exclusively TCA and TCDCA, while the profile in GF *Cyp2c70*^+/+^ mice was dominated by TCA and TβMCA (Figures 4A,B and 5A,B and Supplementary Table S1A–D). Hence, GF mice did not produce secondary bile acids, which is in line with our previous findings [1,25].

HUM *Cyp2c70*^−/−^ mice had a high proportion of TCA and TCDCA in liver and gallbladder, while HUM *Cyp2c70*^+/+^ mice had a high proportion of TCA and TβMCA. Thus, the bile acid profiles in liver and gallbladder of HUM mice were similar to those of GF mice (Figure 4A,B and Supplementary Table S1A,B). In addition to the bile acids found in HUM and GF mice, we found a large proportion of TUDCA in liver and gallbladder of CD *Cyp2c70*^−/−^ mice and of tauro-ω-muricholic acid (TωMCA) in the CD *Cyp2c70*^+/+^ mice (Figure 4A,B and Supplementary Table S1A,B). UDCA and ωMCA can be produced by microbial transformation of CDCA and βMCA respectively [2]. These unconjugated bile acids are then transported to the liver where they can be conjugated to taurine, which is in line with the enrichment of TUDCA and TωMCA in liver and gallbladder of CD mice.

To compare the biochemical properties of the bile acid pools in *Cyp2c70*^−/−^ mice, we calculated the hydrophobicity indices of biliary bile acids in GF, HUM and CD *Cyp2c70*^−/−^ mice. At the early timepoint, there was a slight decrease in the biliary hydrophobicity index in CD *Cyp2c70*^−/−^ females compared to GF *Cyp2c70*^−/−^ females but no difference between the males. However, at the later timepoint the biliary hydrophobicity index was significantly lower in both female and male CD *Cyp2c70*^−/−^ mice compared with their GF and HUM *Cyp2c70*^−/−^ counterparts, largely due to higher amounts of hydrophilic TUDCA, and lower amounts of hydrophobic TCDCA (Figure 4C). The biliary hydrophobicity index of CD *Cyp2c70*^−/−^ mice correlated with relative liver weight (Figure 4D), hepatic gene expression of *Cd177*, *F4/80*, *Tnfα*, and *Ccl2*, and serum levels of AST, and ALT (Supplementary Figure S2), indicating that a more hydrophilic bile acid profile is associated with an improved liver phenotype.

Serum bile acid profiles of HUM mice were dominated by primary bile acids, while CD mice had a more diverse profile including TωMCA/ωMCA in CD *Cyp2c70*^+/+^ mice and TUDCA/UDCA in CD *Cyp2c70*^−/−^ mice (Figure 5A and Supplementary Table S1C). Bile acid profiles in caecum showed that all groups of colonized mice produced the secondary bile acid deoxycholic acid (DCA), which is generated from CA, and both HUM and CD *Cyp2c70*^−/−^ mice, but not *Cyp2c70*^+/+^ mice, produced the secondary bile acid lithocholic acid (LCA), which is generated from CDCA (Figure 5B and Supplementary Table S1D). Additionally, we found the sulphated bile acid CA7S in all groups of colonized mice with particularly high levels in HUM mice (Figure 5B and Supplementary Table S1D), which is in line with our previous observations [25].

*Cyp2c70*^−/−^ mice showed a tendency toward increased total bile acid levels in serum and decreased levels in caecum and gallbladder compared with *Cyp2c70*^+/+^ littermate controls (Supplementary Figure S3 and Supplementary Table S1B–D). This may be interpreted as an indication of decreased biliary bile acid secretion and thus cholestasis, yet, reduced biliary bile acid secretion was not observed in earlier studies [6]. Notably, apart from a small increase in total hepatic bile acid levels in male HUM *Cyp2c70*^−/−^ mice at the early timepoint, deletion of *Cyp2c70* did not affect bile acid levels in the liver, indicating that the liver phenotype of *Cyp2c70*^−/−^ mice was not driven by elevated hepatic bile acid levels (Supplementary Table S1A and Supplementary Figure S3). Noteworthy, the male CD mice had low total bile acid levels in both liver and serum at the later timepoint, without differences between the genotypes (Supplementary Figure S3) indicating normalization of liver function upon conventionalization.

Taken together, we show that deletion of *Cyp2c70* in a GF setting diminished the production of TαMCA and TβMCA and increased the contribution of TCDCA to the bile acid pool. Additionally, *Cyp2c70*^−/−^ mice colonized with human or mouse microbiota generated unconjugated primary bile acids and the secondary bile acids DCA and LCA. Interestingly, colonization with a mouse but not with a human microbiota resulted in increased proportions of TUDCA/UDCA, translating into a more hydrophilic bile acid profile.

### Alterations in the gut microbiota are linked to a more hydrophilic bile acid profile and improved liver phenotype

In line with previous findings that transfer of human microbiota to mice is generally less efficient than transfer of mouse microbiota [25–28], we found that the α-diversity was lower in HUM mice compared with CD mice (Supplementary Figure S4A). The difference in α-diversity was also larger between the human inoculum and HUM recipient mice than between the mouse inoculum and CD recipient mice. Deletion of *Cyp2c70* did not affect α-diversity in neither HUM nor CD mice. Principle coordinate analysis (PCoA) showed that the mouse donor sample clustered together with CD recipient mice while human donor samples and HUM recipient mice were separated, indicating reduced colonization of human microbiota compared with mouse microbiota (Supplementary Figure S4B).

**Figure 6:**
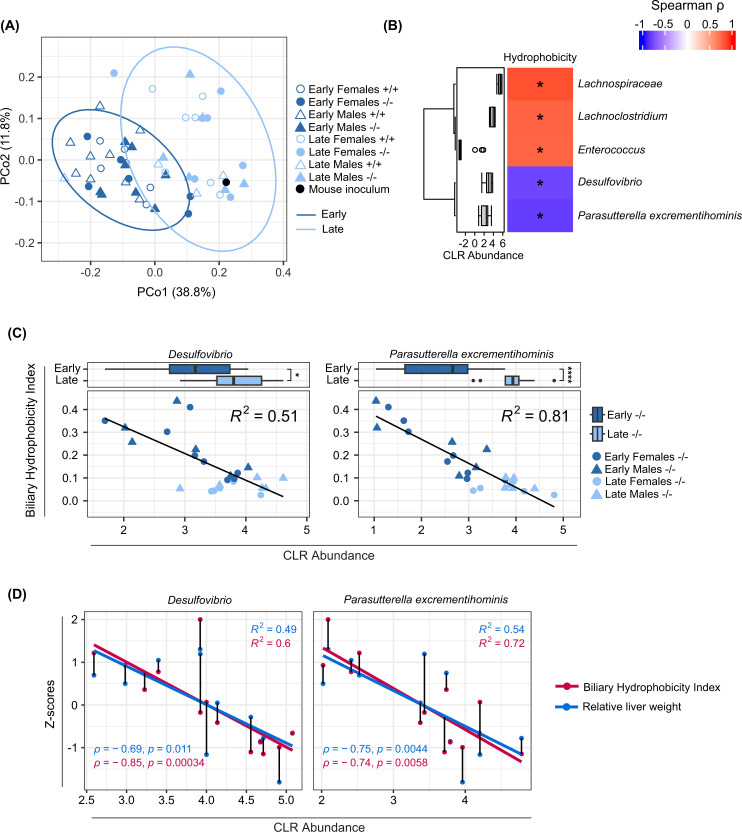
Caecal microbiota analyses in CD mice (**A**) Microbiota analysis by 16S rRNA sequencing of caecum from CD mice showing first and second Principal Coordinates of the weighted UniFrac distance matrix of CD *Cyp2c70*^+/+^ and *Cyp2c70*^−/−^ mice at early and late timepoints and the mouse inoculum. Ellipses indicate 95% confidence assuming a multivariate t-distribution. (**B**) Spearman's correlation between biliary hydrophobicity index and caecal bacterial taxa of CD *Cyp2c70*^−/−^ mice at the early timepoint. Distribution of bacterial abundance is presented as centered log-ratio (CLR) abundance with a box plot. The lower (quartile 1) and upper (quartile 3) quartiles form the box with the median represented by the central line. Whiskers reach to 1.5× the inner quartile range. Outliers are presented with visible points beyond the whiskers. (**C**) Scatterplots with linear models fitted of biliary hydrophobicity index and bacterial taxa presented as CLR abundance for CD *Cyp2c70*^−/−^ mice at early and late timepoints. Distribution of bacterial abundance is presented with a box plot. The lower (quartile 1) and upper (quartile 3) quartiles form the box with the median represented by the central line. Whiskers reach to 1.5× the inner quartile range. Outliers are presented with visible points beyond the whiskers. (**D**) Scatterplots with linear models fitted of bacterial taxa presented as CLR abundance that correlate with biliary hydrophobicity index and relative liver weight in CD *Cyp2c70*^−/−^ mice at the early timepoint. Lines between points signify that the observations are from the same mouse; *P<0.05, **P<0.01, ***P<0.001, ****P<0.0001 indicate differences between *Cyp2c70*^−/−^ mice at early and late timepoints analyzed with Wilcoxon rank sum tests.

We next performed PCoA of the microbiota from CD mice to evaluate differences in composition between genotypes and timepoints and found that the microbiota composition was mainly influenced by timepoint (Figure 6A). However, permutational ANOVA showed that the microbiota composition differed between CD *Cyp2c70*^+/+^ and *Cyp2c70*^−/−^ mice at the early timepoint, and between early and late timepoints in both *Cyp2c70*^+/+^ and *Cyp2c70*^−/−^ mice (Supplementary Table S2A). Hence, there was a genotype-induced difference at the early timepoint. To assess whether there was a link between bile acid profile composition and the gut microbiota, we performed a permutational ANOVA between the biliary hydrophobicity index and the gut microbiota composition in the CD *Cyp2c70*^−/−^ mice at the early timepoint and found that the biliary hydrophobicity index could explain 19.9% of the variation in the gut microbiota composition (Supplementary Table S2B). Next, we performed correlation analyses between the biliary hydrophobicity index and bacterial taxa in CD *Cyp2c70*^−/−^ mice at the early timepoint and found that *Desulfovibrio* and *Parasutterella excrementihomini*s correlated negatively with the biliary hydrophobicity index (Figure 6B). The abundance of these bacterial taxa was higher in the CD *Cyp2c70*^−/−^ mice at the late timepoint, and was associated with a lower hydrophobicity index (Figure 6C). Finally, a more hydrophilic bile acid profile, as well as a lower relative liver weight was associated with increased abundance of both *Desulfovibrio* and *P. excrementihominis* (Figure 6D).

## Discussion

In this study we used the novel *Cyp2c70*^−/−^ mouse model to investigate whether a gut microbiota can be protective in the development of hepatobiliary disease. We rederived *Cyp2c70*^−/−^ mice as GF and demonstrated that deletion of *Cyp2c70* in the absence of bacteria led to a markedly reduced neonatal survival. To examine the protective role of the gut microbiota, we colonized GF *Cyp2c70*^+/−^ mice with either a human or a mouse microbiota and showed that the presence of a microbiota improved neonatal survival of *Cyp2c70*^−/−^ mice, independent of donor origin, while signs of liver fibrosis, immune cell infiltration and expression of inflammatory markers as well as ductular reactions only improved in *Cyp2c70*^−/−^ mice colonized with a mouse microbiota.

The increased mortality of GF *Cyp2c70*^−/−^ mice may be caused by the substantially elevated levels of the hydrophobic, and thus cytotoxic, bile acid TCDCA [14]. TCDCA cannot be metabolized in a GF setting and this could lead to toxic effects during the neonatal state. However, our bile acid analyses showed that bile acid profiles in liver and gallbladder were very similar in HUM and GF *Cyp2c70*^−/−^ mice, yet neonatal survival was improved in the HUM *Cyp2c70*^−/−^ mice. This suggests that there are other developmental changes occurring after birth, which are microbiota-dependent and could be crucial for coping with toxic bile acids, such as formation of bile ducts or structures in the gastrointestinal tract [29–31].

HUM *Cyp2c70*^−/−^ mice had decreased microbial diversity compared with CD *Cyp2c70*^−/−^ mice. Still the bile acid profile in caecum was rather similar in HUM and CD *Cyp2c70*^−/−^ mice, indicating that the decreased diversity was not associated with a decreased capacity to produce secondary bile acids in general. On the contrary, bile acid profiles in liver and gallbladder differed substantially between HUM and CD *Cyp2c70*^−/−^ mice. CD *Cyp2c70*^−/−^ mice had a large proportion of TUDCA, while HUM and GF *Cyp2c70*^−/−^ mice had similar profiles dominated by TCDCA and TCA. A small proportion of TUDCA was detected in the GF mice, which confirms our previous finding that UDCA is a primary bile acid in mice [1]. However, there was a robust increase in TUDCA and UDCA production in the CD mice suggesting that UDCA primarily is a secondary bile acid produced by specific bacteria. The most likely route for UDCA production is 7β-epimerization from CDCA via the intermediate 7-oxoCDCA [32,33]. However, we cannot determine whether all steps in the transformation were carried out by bacteria in the gut or if some modifications occurred in the liver. It has previously been shown in an *in vitro* study using mouse liver microsomes that 7-oxoCDCA can be converted to CDCA and, to a lesser extent, to UDCA [34]. Interestingly, the levels of UDCA were low in caecum suggesting that UDCA was produced in the upper part of the gut and efficiently absorbed into the enterohepatic circulation, or alternatively, that it was produced in the liver. The HUM *Cyp2c70*^−/−^ mice had high levels of the precursor CDCA yet very low UDCA levels, indicating that some microbial enzymes essential for 7β-epimerization of CDCA were missing in the HUM *Cyp2c70*^−/−^ mice. This could be due to lack of specific bacteria in the human donor sample or due to limited transfer of human bacteria into the recipient mice, which has been reported in previous studies with GF or antibiotic-treated wild-type mice [25–28]. We hypothesized that colonization of *Cyp2c70*^−/−^ mice with a human microbiota, which have a human-like bile acid profile, would be a more physiological model to study bile acid–microbiota interactions in liver disease. However, the reduced neonatal survival of GF *Cyp2c70*^−/−^ mice prohibited us from colonizing adult or adolescent GF *Cyp2c70*^−/−^ mice and instead we colonized heterozygous breeding pairs, which is a limitation of the model. If *Cyp2c70*^−/−^ mice could be colonized directly, the transfer of human bacteria might be improved, and it would also facilitate colonization and comparison between different human donors.

The proportion of TUDCA/UDCA was increased at the later timepoint, which was reflected by a decreased biliary hydrophobicity index, and we believe that the improved hepatobiliary phenotypes in CD *Cyp2c70*^−/−^ mice at this timepoint could be, at least in part, attributed to microbial production of hydrophilic, and thus hepatoprotective, UDCA. Indeed, feeding of female *Cyp2c70*^−/−^ mice with a UDCA-enriched diet (0.1% wt/wt) fully restored cholangiopathy within a 6-week time frame [6], while the hydrophobic FXR agonist obeticholic acid (OCA) was not effective [35]. We found that a lower hydrophobicity index was associated with lower relative liver weight, and that this was associated with increased abundance of *Desulfovibrio* and *P. excrementihominis*. However, there is no evidence that these are UDCA-producing bacteria.

In accordance with our results, previous studies on the cholestatic mouse model induced by deletion of *Mdr2/Abcb4*, have also demonstrated a worsened hepatobiliary phenotype in GF conditions, suggesting that a commensal microbiota may be protective against bile duct injury [9,36]. One important difference between the *Mdr2*^−/−^ mouse model and our model, is that *Mdr2*^−/−^ mice have a murine primary bile acid profile but lack phospholipids in bile [37], while *Cyp2c70*^−/−^ mice have a human-like primary bile acid profile, which is more hydrophobic, and hence toxic, and a higher biliary phospholipid content [6]. Interestingly, one study found no increased mortality in GF *Mdr2*^−/−^ mice during the first 60 days [9], while another study found no neonatal mortality, but 100% mortality at 8 weeks. Furthermore, treatment with broad spectrum antibiotics in adult *Mdr2*^−/−^ mice, which result in a GF-like condition, increased the lethality [10].

One common feature of the *Mdr2*^−/−^ and *Cyp2c70*^−/−^ models is that UDCA seems to be protective against the somewhat similar hepatobiliary phenotypes. Previous studies have shown that UDCA feeding reverses the adult-onset of a cholestatic liver phenotype in conventionally raised *Cyp2c70*^−/−^ mice at 27 weeks of age [6] and improves liver pathology in *Mdr2*^−/−^ mice [38,39]. Another study showed that perinatal exposure to UDCA prevented neonatal cholestasis of conventionally raised *Cyp2c70*^−/−^ mice, but there were no long-lasting effects on liver pathophysiology after discontinuation of UDCA treatment [40].

In line with our findings that a gut microbiota has a protective role in hepatobiliary disease, colonization experiments with GF *Mdr2*^−/−^ mice showed that liver-related mortality was reduced when mice were colonized at 3–4 weeks of age, but not when colonization was performed at 5–7 weeks [36]. However, additional experiments identified some bacteria, such as *Enterococcus faecalis* and *Escherichia coli*, with detrimental effects on disease development, which underscores that the role of the microbiota in hepatobiliary disease is complex and needs further investigation.

In conclusion, we show that the presence of a gut microbiota is important for neonatal survival of *Cyp2c70*^−/−^ mice. We also show that microbially induced production of TUDCA/UDCA results in a hydrophilic bile acid profile, which correlates with improved liver phenotype in *Cyp2c70*^−/−^ mice. Our model emphasizes that there is an important link between the gut microbiota and hepatobiliary disease and a future strategy could be to identify UDCA-producing bacteria and test their hepatoprotective ability.

## Clinical perspectives

- Conventionally raised Cyp2c70^−/−^ mice with a human-like bile acid composition have shown promise as a model for hepatobiliary disease, however, the impact of the gut microbiota in disease development and progression is unknown.
- We rederived Cyp2c70^−/−^ mice as GF and showed that the absence of a gut microbiota reduced neonatal survival, and that colonization with a human or mouse microbiota improved neonatal survival while only mouse microbiota induced TUDCA/UDCA production, which was reflected by a lower hydrophobicity index of biliary bile acids and improved liver phenotype.
- Our findings indicate that microbially induced production of TUDCA/UDCA may protect against hepatobiliary disease in Cyp2c70^−/−^ mice.

## Supplementary Material

Supplementary Figures S1-S4

Supplementary Tables S1-S2

## Data Availability

16S rRNA sequencing data have been deposited to the European Nucleotide Archive under accession number PRJEB61888 (https://www.ebi.ac.uk/ena/browser/view/PRJEB61888). The random forest model for Sirius Red histological analysis is available at Github (https://github.com/wilhelmsjoland/Cyp2c70).

## Open Access

Open access for this article was enabled by the participation of University Of Gothenburg in an all-inclusive *Read & Publish* agreement with Portland Press and the Biochemical Society under a transformative agreement with Individual.

## Abbreviations

αMCA: α-muricholic acid
βMCA: β-muricholic acid
ωMCA: ω-muricholic acid
7-oxoCA: 7-oxocholic acid
7-oxoCDCA: 7-oxochenodeoxycholic acid
CA7S: cholic acid-7-sulfate
CA: cholic acid
CDCA: chenodeoxycholic acid
DCA: deoxycholic acid
HDCA: hyodeoxycholic acid
isoDCA: iso-deoxycholic acid (3β-deoxycholic acid)
isoLCA: iso-lithocholic acid (3β-lithocholic acid)
LCA: lithocholic acid
TαMCA: tauro-α-muricholic acid
TβMCA: tauro-β-muricholic acid
TωMCA: tauro-ω-muricholic acid
TCA: tauro-cholic acid
TCDCA: tauro-chenodeoxycholic acid
TDCA: tauro-deoxycholic acid
THCA: tauro-hyocholic acid
TUDCA: tauro-ursodeoxycholic acid
UDCA: ursodeoxycholic acid

## References

[1] Sayin S.I., Wahlstrom A., Felin J., Jantti S., Marschall H.U., Bamberg K. et al. (2013) Gut microbiota regulates bile acid metabolism by reducing the levels of tauro-beta-muricholic acid, a naturally occurring FXR antagonist. Cell Metab. 17, 225–235 10.1016/j.cmet.2013.01.003 23395169

[2] Wahlstrom A., Sayin S.I., Marschall H.U. and Backhed F. (2016) Intestinal crosstalk between bile acids and microbiota and its impact on host metabolism. Cell Metab. 24, 41–50 10.1016/j.cmet.2016.05.005 27320064

[3] Takahashi S., Fukami T., Masuo Y., Brocker C.N., Xie C., Krausz K.W. et al. (2016) Cyp2c70 is responsible for the species difference in bile acid metabolism between mice and humans. J. Lipid Res. 57, 2130–2137 10.1194/jlr.M071183 27638959 PMC5321228

[4] Honda A., Miyazaki T., Iwamoto J., Hirayama T., Morishita Y., Monma T. et al. (2020) Regulation of bile acid metabolism in mouse models with hydrophobic bile acid composition. J. Lipid Res. 61, 54–69 10.1194/jlr.RA119000395 31645370 PMC6939601

[5] de Boer J.F., Verkade E., Mulder N.L., de Vries H.D., Huijkman N., Koehorst M. et al. (2020) A human-like bile acid pool induced by deletion of hepatic Cyp2c70 modulates effects of FXR activation in mice. J. Lipid Res. 61, 291–305 10.1194/jlr.RA119000243 31506275 PMC7053831

[6] de Boer J.F., de Vries H.D., Palmiotti A., Li R., Doestzada M., Hoogerland J.A. et al. (2021) Cholangiopathy and biliary fibrosis in Cyp2c70-deficient mice are fully reversed by ursodeoxycholic acid. Cell Mol. Gastroenterol. Hepatol. 11, 1045–1069 10.1016/j.jcmgh.2020.12.004 33309945 PMC7898074

[7] Tabibian J.H., Talwalkar J.A. and Lindor K.D. (2013) Role of the microbiota and antibiotics in primary sclerosing cholangitis. Biomed. Res. Int. 2013, 389537 10.1155/2013/389537 24232746 PMC3819830

[8] Hov J.R. and Karlsen T.H. (2023) The microbiota and the gut-liver axis in primary sclerosing cholangitis. Nat. Rev. Gastroenterol. Hepatol. 20, 135–154 10.1038/s41575-022-00690-y 36352157

[9] Tabibian J.H., O'Hara S.P., Trussoni C.E., Tietz P.S., Splinter P.L., Mounajjed T. et al. (2016) Absence of the intestinal microbiota exacerbates hepatobiliary disease in a murine model of primary sclerosing cholangitis. Hepatology 63, 185–196 10.1002/hep.27927 26044703 PMC4670294

[10] Schneider K.M., Candels L.S., Hov J.R., Myllys M., Hassan R., Schneider C.V. et al. (2021) Gut microbiota depletion exacerbates cholestatic liver injury via loss of FXR signalling. Nat. Metab. 3, 1228–1241 10.1038/s42255-021-00452-1 34552267

[11] Marschall H.U. and Backhed F. (2016) Could gut microbiota protect against sclerosing cholangitis? Hepatology 63, 26–27 10.1002/hep.28135 26315989

[12] Liang W., Menke A.L., Driessen A., Koek G.H., Lindeman J.H., Stoop R. et al. (2014) Establishment of a general NAFLD scoring system for rodent models and comparison to human liver pathology. PLoS ONE 9, ARTN e115922 10.1371/journal.pone.0115922 25535951 PMC4275274

[13] Yaniv Z., Lowekamp B.C., Johnson H.J. and Beare R. (2019) Correction to: SimpleITK image-analysis notebooks: a collaborative environment for education and reproducible research. J. Digit. Imaging 32, 1118 10.1007/s10278-018-0165-9 31485952 PMC6841876

[14] Heuman D.M. (1989) Quantitative estimation of the hydrophilic-hydrophobic balance of mixed bile salt solutions. J. Lipid Res. 30, 719–730 10.1016/S0022-2275(20)38331-0 2760545

[15] Kozich J.J., Westcott S.L., Baxter N.T., Highlander S.K. and Schloss P.D. (2013) Development of a dual-index sequencing strategy and curation pipeline for analyzing amplicon sequence data on the MiSeq Illumina sequencing platform. Appl. Environ. Microbiol. 79, 5112–5120 10.1128/AEM.01043-13 23793624 PMC3753973

[16] Edgar R.C. (2010) Search and clustering orders of magnitude faster than BLAST. Bioinformatics 26, 2460–2461 10.1093/bioinformatics/btq461 20709691

[17] Edgar R.C. (2016) UNOISE2: improved error-correction for Illumina 16S and ITS amplicon sequencing. bioRxiv 081257 10.1101/081257

[18] Wang Q., Garrity G.M., Tiedje J.M. and Cole J.R. (2007) Naive Bayesian classifier for rapid assignment of rRNA sequences into the new bacterial taxonomy. Appl. Environ. Microbiol. 73, 5261–5267 10.1128/AEM.00062-07 17586664 PMC1950982

[19] Callahan B.J., McMurdie P.J., Rosen M.J., Han A.W., Johnson A.J. and Holmes S.P. (2016) DADA2: High-resolution sample inference from Illumina amplicon data. Nat. Methods 13, 581–583 10.1038/nmeth.3869 27214047 PMC4927377

[20] Yilmaz P., Parfrey L.W., Yarza P., Gerken J., Pruesse E., Quast C. et al. (2014) The SILVA and “All-species Living Tree Project (LTP)” taxonomic frameworks. Nucleic Acids Res. 42, D643–D648 10.1093/nar/gkt1209 24293649 PMC3965112

[21] Quast C., Pruesse E., Yilmaz P., Gerken J., Schweer T., Yarza P. et al. (2013) The SILVA ribosomal RNA gene database project: improved data processing and web-based tools. Nucleic Acids Res. 41, D590–D596 10.1093/nar/gks1219 23193283 PMC3531112

[22] McMurdie P.J. and Holmes S. (2013) phyloseq: an R package for reproducible interactive analysis and graphics of microbiome census data. PLoS ONE 8, e61217 10.1371/journal.pone.0061217 23630581 PMC3632530

[23] Oksanen J., Blanchet F.G., Kindt R., Legendre P., Minchin P.R., O'hara R.B. et al. (2013) Community ecology package. R Package Version 2, 321–326

[24] Truong J.K., Bennett A.L., Klindt C., Donepudi A.C., Malla S.R., Pachura K.J. et al. (2022) Ileal bile acid transporter inhibition in Cyp2c70 KO mice ameliorates cholestatic liver injury. J. Lipid Res. 63, 100261 10.1016/j.jlr.2022.100261 35934110 PMC9460185

[25] Wahlstrom A., Kovatcheva-Datchary P., Stahlman M., Khan M.T., Backhed F. and Marschall H.U. (2017) Induction of farnesoid X receptor signaling in germ-free mice colonized with a human microbiota. J. Lipid Res. 58, 412–419 10.1194/jlr.M072819 27956475 PMC5282957

[26] Turnbaugh P.J., Ridaura V.K., Faith J.J., Rey F.E., Knight R. and Gordon J.I. (2009) The effect of diet on the human gut microbiome: a metagenomic analysis in humanized gnotobiotic mice. Sci. Transl. Med. 1, 6ra14 10.1126/scitranslmed.3000322 20368178 PMC2894525

[27] Staley C., Kaiser T., Beura L.K., Hamilton M.J., Weingarden A.R., Bobr A. et al. (2017) Stable engraftment of human microbiota into mice with a single oral gavage following antibiotic conditioning. Microbiome 5, 87 10.1186/s40168-017-0306-2 28760163 PMC5537947

[28] Zhang L., Bahl M.I., Roager H.M., Fonvig C.E., Hellgren L.I., Frandsen H.L. et al. (2017) Environmental spread of microbes impacts the development of metabolic phenotypes in mice transplanted with microbial communities from humans. ISME J. 11, 676–690 10.1038/ismej.2016.151 27858930 PMC5322303

[29] Tanimizu N., Kaneko K., Itoh T., Ichinohe N., Ishii M., Mizuguchi T. et al. (2016) Intrahepatic bile ducts are developed through formation of homogeneous continuous luminal network and its dynamic rearrangement in mice. Hepatology 64, 175–188 10.1002/hep.28521 26926046

[30] Lemaigre F.P. (2003) Development of the biliary tract. Mech. Dev. 120, 81–87 10.1016/S0925-4773(02)00334-9 12490298

[31] Frazer L.C. and Good M. (2022) Intestinal epithelium in early life. Mucosal. Immunol. 15, 1181–1187 10.1038/s41385-022-00579-8 36380094 PMC10329854

[32] Salen G., Verga D., Batta A.K., Tint G.S. and Shefer S. (1982) Effect of 7-ketolithocholic acid on bile acid metabolism in humans. Gastroenterology 83, 341–347 10.1016/S0016-5085(82)80326-0 7084613

[33] Lee J.Y., Arai H., Nakamura Y., Fukiya S., Wada M. and Yokota A. (2013) Contribution of the 7beta-hydroxysteroid dehydrogenase from Ruminococcus gnavus N53 to ursodeoxycholic acid formation in the human colon. J. Lipid Res. 54, 3062–3069 10.1194/jlr.M039834 23729502 PMC3793610

[34] Penno C.A., Morgan S.A., Vuorinen A., Schuster D., Lavery G.G. and Odermatt A. (2013) Impaired oxidoreduction by 11beta-hydroxysteroid dehydrogenase 1 results in the accumulation of 7-oxolithocholic acid. J. Lipid Res. 54, 2874–2883 10.1194/jlr.M042499 23933573 PMC3770100

[35] Li R., Hovingh M.V., Koehorst M., de Blaauw P., Verkade H.J., de Boer J.F. et al. (2022) Short-term obeticholic acid treatment does not impact cholangiopathy in Cyp2c70-deficient mice with a human-like bile acid composition. Biochim. Biophys. Acta Mol. Cell. Biol. Lipids 1867, 159163 10.1016/j.bbalip.2022.159163 35470044

[36] Awoniyi M., Wang J., Ngo B., Meadows V., Tam J., Viswanathan A. et al. (2023) Protective and aggressive bacterial subsets and metabolites modify hepatobiliary inflammation and fibrosis in a murine model of PSC. Gut 72, 671–685 10.1136/gutjnl-2021-326500 35705368 PMC9751228

[37] Smit J.J., Schinkel A.H., Oude Elferink R.P., Groen A.K., Wagenaar E., van Deemter L. et al. (1993) Homozygous disruption of the murine mdr2 P-glycoprotein gene leads to a complete absence of phospholipid from bile and to liver disease. Cell 75, 451–462 10.1016/0092-8674(93)90380-9 8106172

[38] Van Nieuwkerk C.M., Elferink R.P., Groen A.K., Ottenhoff R., Tytgat G.N., Dingemans K.P. et al. (1996) Effects of Ursodeoxycholate and cholate feeding on liver disease in FVB mice with a disrupted mdr2 P-glycoprotein gene. Gastroenterology 111, 165–171 10.1053/gast.1996.v111.pm8698195 8698195

[39] Meng F., Kennedy L., Hargrove L., Demieville J., Jones H., Madeka T. et al. (2018) Ursodeoxycholate inhibits mast cell activation and reverses biliary injury and fibrosis in Mdr2(-/-) mice and human primary sclerosing cholangitis. Lab. Invest. 98, 1465–1477 10.1038/s41374-018-0101-0 30143751 PMC6214746

[40] de Vries H.D., Palmiotti A., Li R., Hovingh M.V., Mulder N.L., Koehorst M. et al. (2023) Perinatal exposure to UDCA prevents neonatal cholestasis in Cyp2c70(-/-) mice with human-like bile acids. Pediatr. Res. 93, 1582–1590 10.1038/s41390-022-02303-5 36151295 PMC10172110

