# Supplementary material for: Absence of gut microbiota reduces neonatal survival and exacerbates liver disease in Cyp2c70-deficient mice with a human-like bile acid composition
Source: Clin Sci (Lond). 2023 Jul 14;137(13):995–1011. doi: 10.1042/CS20230413 (PMC10346113; doi:10.1042/CS20230413)
Supplement: Supplementary Figures S1-S4 [file CS-2023-0413_supp.pdf]

Supplementary Figure 1

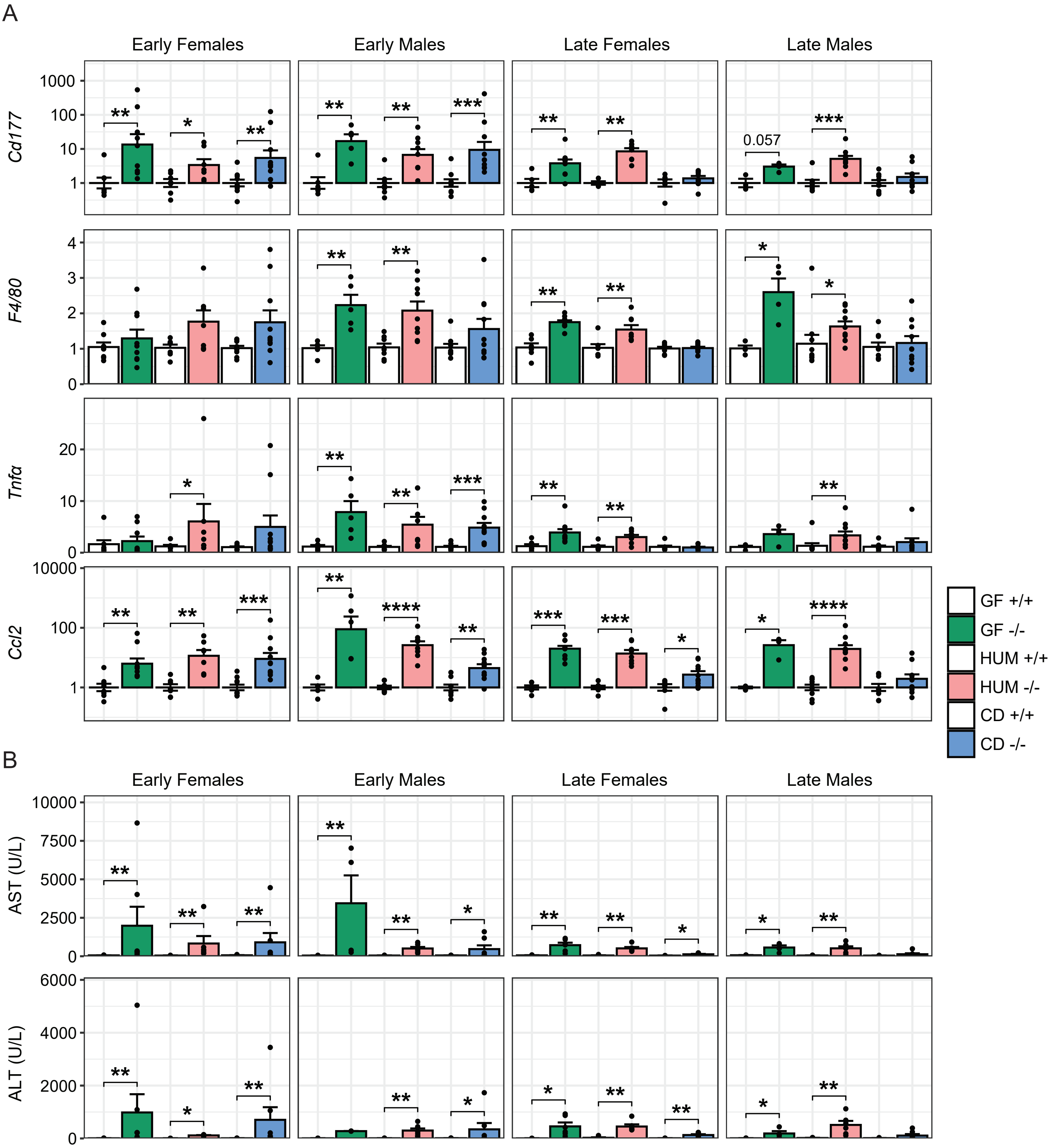

Supplementary Figure 1. Attenuation of hepatic immune cell infiltration, inflammation, and liver transaminases in CD *Cyp2c70*-/- mice at the late timepoint. (A) Relative gene expression of hepatic *Cd177*, *F4/80*, *Tnfα*, and *Ccl2* in GF, HUM, and CD *Cyp2c70*+/+ and *Cyp2c70*-/- females and males at early and late timepoints. (n = 4-10 mice per group). (B) Serum levels of liver transaminases, aspartate aminotransferase (AST; U/L), alanine aminotransferase (ALT; U/L), in GF, HUM, and CD *Cyp2c70*+/+ and *Cyp2c70*-/- females and males at early and late timepoints. (n = 4-7 mice per group). Data are presented as mean ± SEM, * P < 0.05, ** P < 0.01, *** P < 0.001, **** P < 0.0001 indicate differences between female or male *Cyp2c70*+/+ and *Cyp2c70*-/- mice within each group (GF, HUM or CD) analyzed with Wilcoxon rank sum tests.

Supplementary Figure 2

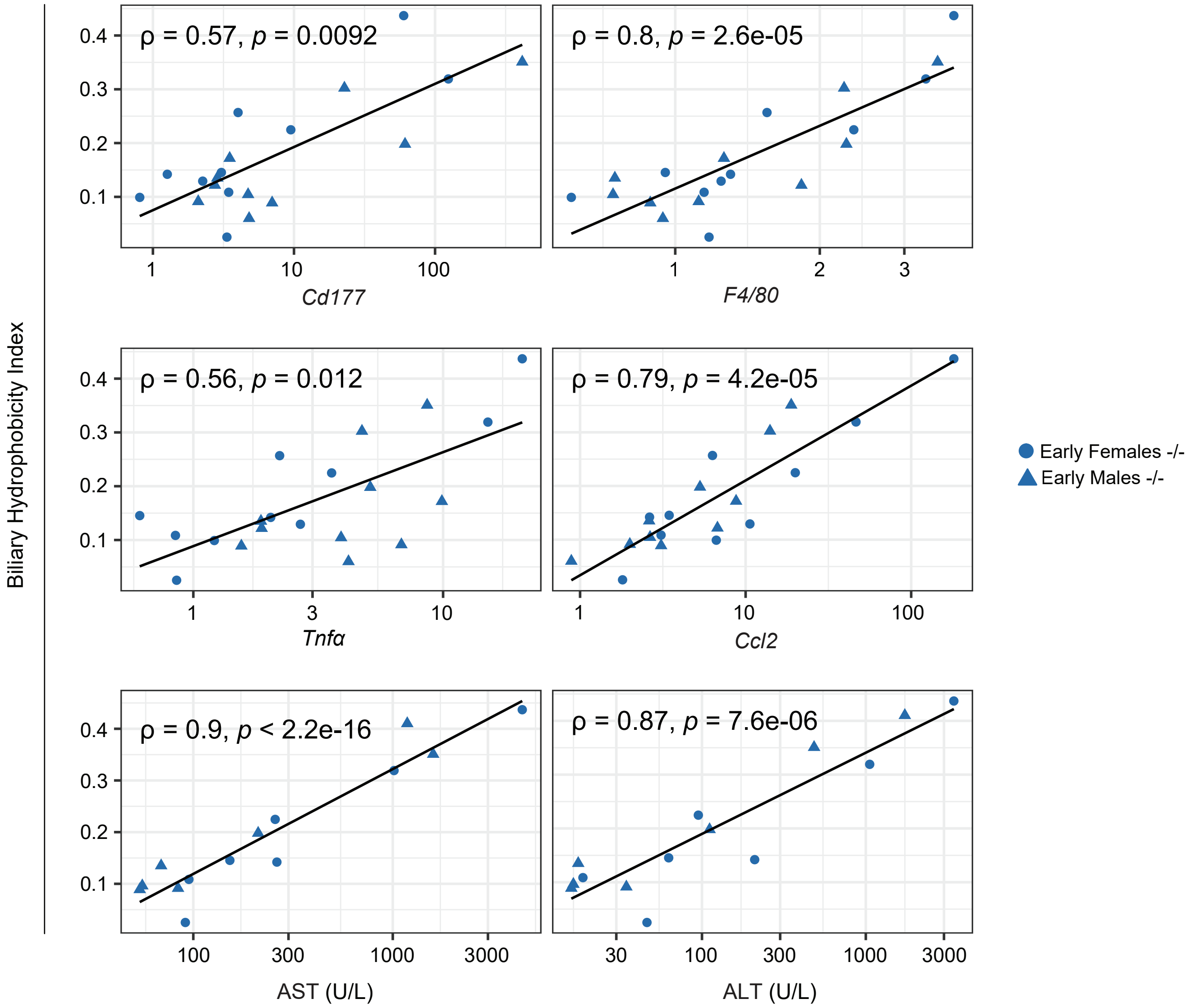

Supplementary Figure 2. Hydrophobicity index of biliary bile acids correlates with markers of hepatic immune cell infiltration, inflammation, and liver transaminases in CD *Cyp2c70*$^{-/-}$ mice at the early timepoint. Scatterplots with a linear model fit to the log10 x-axis and Spearman's correlation analysis of biliary hydrophobicity index and relative expression of *Cd177*, *F4/80*, *Tnfα*, and *Ccl2* (n = 20), and serum levels of aspartate aminotransferase (AST; U/L) and alanine aminotransferase (ALT; U/L) (n = 14) in CD *Cyp2c70*$^{-/-}$ females and males at the early timepoint.

Supplementary Figure 3

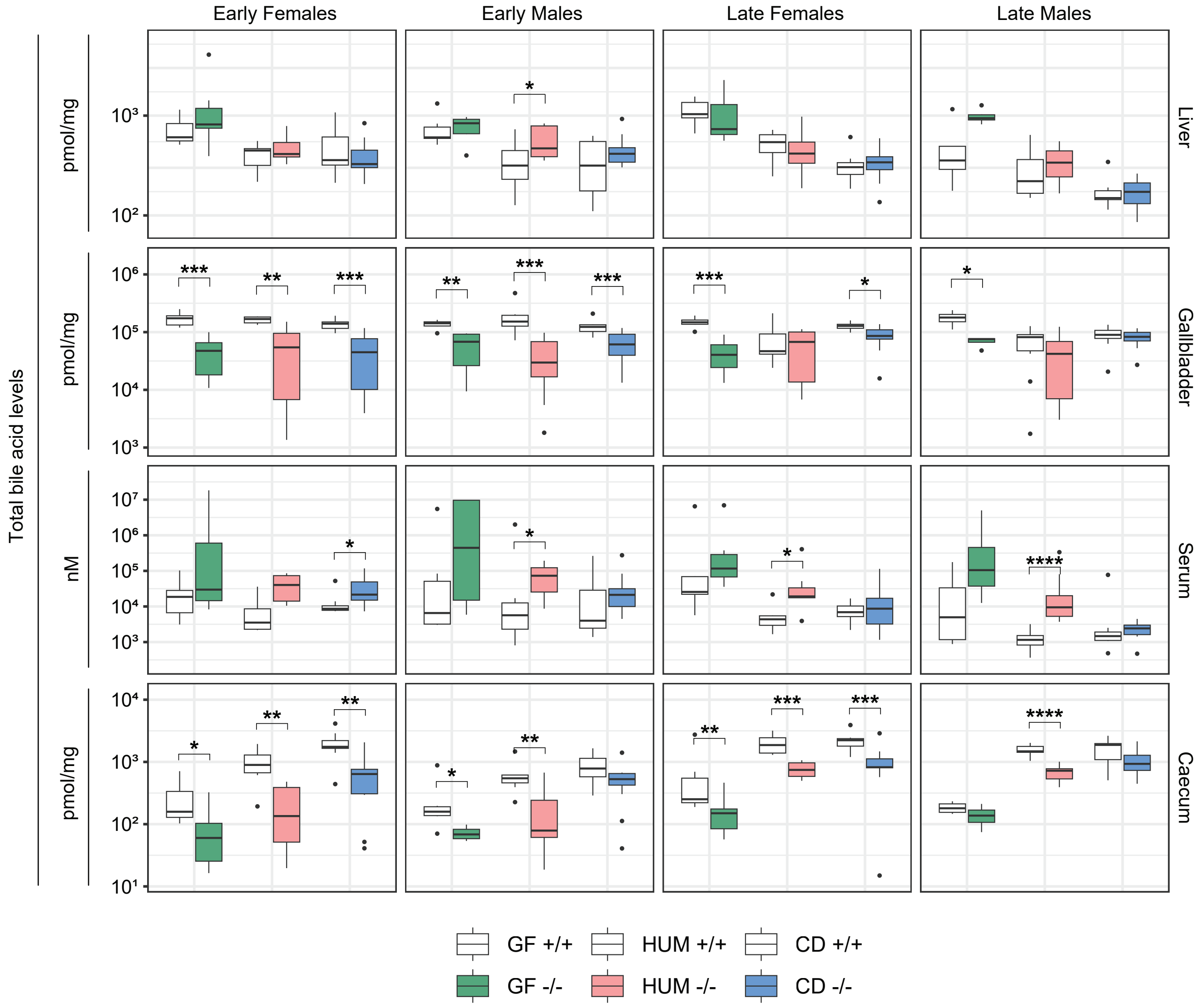

Supplementary Figure 3. Total amount of bile acids in liver, gallbladder, serum and caecum. Total amount of bile acids in liver, gallbladder, serum, and caecum of *Cyp2c70*$^{+/+}$ and *Cyp2c70*$^{-/-}$ mice presented with a box plot. The lower (quartile 1) and upper (quartile 3) quartiles form the box with the median represented by the central line. Whiskers reach to 1.5X the inner quartile range. Outliers are presented with visible points beyond the whiskers. * $P < 0.05$, ** $P < 0.01$, *** $P < 0.001$, **** $P < 0.0001$ indicate differences between female or male *Cyp2c70*$^{+/+}$ and *Cyp2c70*$^{-/-}$ mice within each group (GF, HUM or CD) analyzed with Wilcoxon rank sum tests.

Supplementary Figure 4

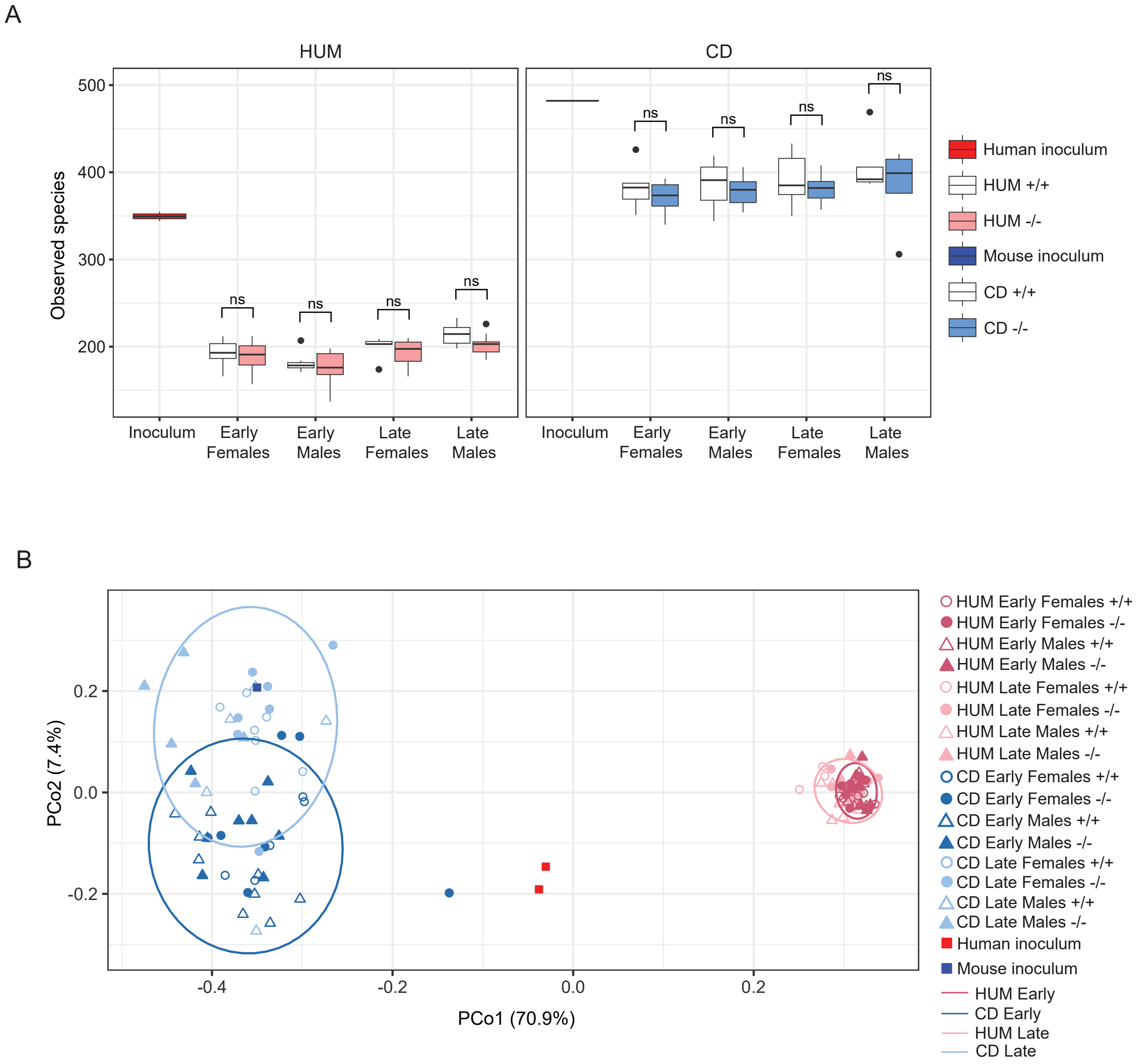

Supplementary Figure 4. Microbiota composition in HUM and CD *Cyp2c70* mice.
(A) Caecal microbial composition of CD and HUM *Cyp2c70*$^{+/+}$ and *Cyp2c70*$^{-/-}$ mice and their corresponding inocula presented by α-diversity described as observed species in a box plot. The lower (quartile 1) and upper (quartile 3) quartiles form the box with median represented by the central line. Whiskers reach to 1.5X the inner quartile range. Outliers are presented with visible points beyond the whiskers. (B) Caecal microbial composition of CD and HUM *Cyp2c70*$^{+/+}$ and *Cyp2c70*$^{-/-}$ mice and their corresponding inocula presented by β-diversity described by PCoA showing first and second Principal Coordinates of the weighted UniFrac distance matrix.
